# A Review of Basic Knowledge of HIV Infection for Orthodontic Management of HIV Patients

**DOI:** 10.7759/cureus.37770

**Published:** 2023-04-18

**Authors:** Saritha Madham, J Visshishta, Haritha Dasagari Vinod, Ojass Kumar S, Vishnu Priya Cherukuri

**Affiliations:** 1 Orthodontics and Dentofacial Orthopaedics, Malla Reddy Institute of Dental Sciences, Hyderabad, IND; 2 Orthodontics and Dentofacial Orthopaedics, AH Dental and Orthodontic Centre, Hyderabad, IND; 3 Orthodontics and Dentofacial Orthopaedics, Malla Reddy Dental College for Women, Hyderabad, IND; 4 Orthodontics and Dentofacial Orthopaedics, MNR Dental College & Hospital, Hyderabad, IND

**Keywords:** hiv transmission, post exposure prophylaxis, orthodontic management, aids, hiv

## Abstract

Human immunodeficiency virus (HIV) is a retrovirus that causes an infection affecting immunity-providing white blood cells. The HIV pandemic is far from over and is a major socio-economical burden. As there is no cure yet, prevention of new infections is the major path to control the infection. There is a low chance of orthodontic procedures carrying a risk of HIV infection transmission. It is important to have knowledge about the disease to effectively and safely treat known or unknown patients with HIV.

## Introduction and background

Human immunodeficiency virus (HIV) is a retrovirus that causes an infection affecting immunity-providing white blood cells. HIV destroys the CD4 cells, weakening a person’s immunity against opportunistic infections, such as tuberculosis and fungal infections, severe bacterial infections and some cancers [1]. Acquired immunodeficiency syndrome (AIDS) is a term that applies to the most advanced stages of HIV infection. It is defined by the occurrence of any of the more than 20 life-threatening cancers or “opportunistic infections” (OI), so named because they take advantage of a weakened immune system [2].

African countries have the major load of HIV cases with many young adults positive for infection. Antiretroviral treatment (ART) has made a tremendous difference in AIDS-related deaths and the transmission risk; however, ART is still not accessible universally. The development of a cure or an effective vaccine is still under trial. Therefore, HIV/AIDS will take a huge toll on the world in near future [3].

With improvement in quality of life from ART, orthodontists will see an increased number of HIV-positive people seeking orthodontic treatment. Current literature shows that orthodontists still feel that they require more information on orthodontic care in HIV-infected people to be able to treat them confidently [4]. Therefore, HIV-specific knowledge and training will benefit orthodontists.

Origins of HIV

The HIV virus is considered to have evolved because of the interspecies spread of slow-type retroviruses that are native to different non-human primates. African primates like African green monkeys and chimpanzees showed distinct simian immunodeficiency viruses (SIVs) that were non-pathogenic in their natural hosts. Two types of HIV viruses have been identified. HIV-1, which is the most widespread strain, has been perceived to originate from SIVcpz present in chimpanzees. HIV-2 shows a resemblance to SIV which causes immune deficiency in macaques and is confined to the West African region. HIV-2 originating from sooty mangabey was confirmed by demonstrating a resemblance to locally circulating SIVsmm infections in West Africa [3].

History and epidemiology

In 1981, many young homosexual men were dying because of unusual and rare infections and malignancies with unknown causes. These collections of conditions were acknowledged to be of the same origin and were defined as acquired immune deficiency syndrome (AIDS). The AIDS-causing virus was identified later in 1983 [3].

So far, 84.2 million people have become infected with HIV and 40.1 million have died from AIDS-related illnesses. According to a UNAIDS factsheet, globally, an estimated 38.4 million people were living with HIV in 2021, of which 1.5 million were newly infected. Around 85% of all people living with HIV (PLHIV) knew their HIV status and about 5.9 million people did not know that they were living with HIV in 2021. Worldwide, 28.7 million (75%) of PLHIV were accessing treatment. Focus on the prevention of HIV infection and access to treatment is helping to control the pandemic but there is still a long way to go to reach the goal of 95-95-95 by 2025. New HIV infections have reduced by 54% since the peak in 1996. AIDS-related deaths have reduced by 68% since the peak in 2004 and by 52% since 2010 [5].

**Figure 1:**
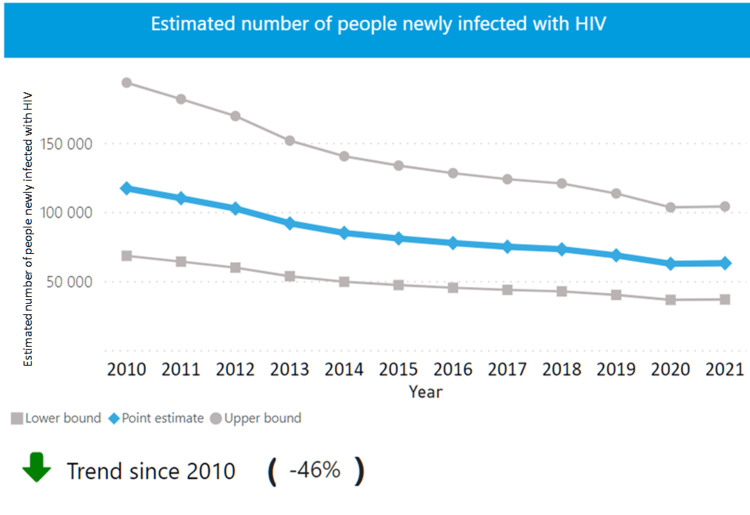
New HIV cases in India from 2010 to 2021 Source: India - HIV country profile 2022. UNAIDS/WHO 2022 estimates [8].

**Figure 2:**
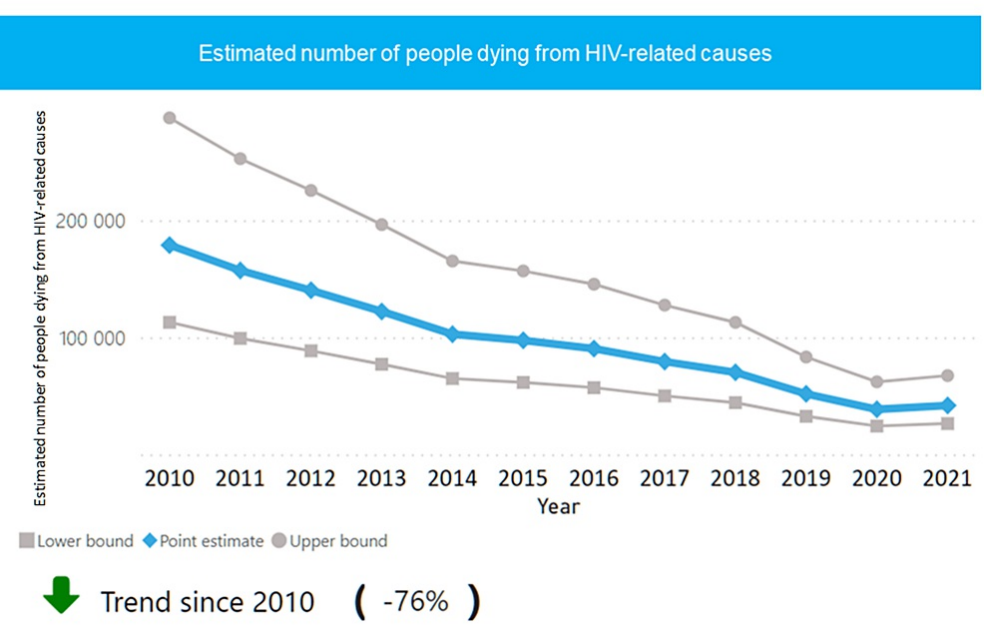
HIV-related deaths in India between 2010 and 2021 Source: India - HIV country profile 2022. UNAIDS/WHO 2022 estimates [8].

In 1986, India started conducting HIV screening amongst high-risk groups and thus HIV was first detected in samples of female sex workers from Chennai. This led to the formation of the National AIDS Committee in 1986 and the National AIDS Control Organization (NACO) in 1992 which provide guidelines on HIV prevention and management in India [6]. In India, 2,400,000 people were living with HIV in 2021, with 63,000 new cases. Around 77% PLHIVs knew their status and 65% were receiving ART and 42,000 AIDS-related deaths were noted [7]. India also noted a downward trend in newly infected people and in HIV-related deaths since 2010 as seen in Figures 1-2 [8]. Based on the count of people, India stands third in the world in HIV infections, with around 2,319,000 people affected in 2020 [6].

## Review

Transmission

Molecular studies have predicted that the cross-species jump to humans may have happened around 1880 to 1940 in Africa yet the first case was diagnosed in the 1980s. It is thought that the early spread and adaptation of the HIV virus could have been because of the reuse of syringes and needles during medical and pharmaceutical campaigns in poor African nations in the 1900s, accompanied by destabilization of social structures, urbanization and increased mobility, and an uptick in sexually transmitted diseases [3,9].

HIV is transmitted by the exchange of a variety of body fluids from infected people, such as blood, breast milk, semen and vaginal secretions [2]. Heterosexual transmission is still the dominant mode of transmission [10].

Routes of Transmission of HIV

Medical/iatrogenic transmission involves blood and blood product transfusions and related activities, injections, and other invasive procedures: surgery, dialysis, dental work, colonoscopy, and so on. Nonmedical transmission occurs via intravenous drug abuse, homosexual relations, heterosexual relations, vertical transmission (mother to child in utero or breastfeeding), tattoos, body piercing, scarifications, genital mutilation, and so on [9].

The efficiency of transmission is also defined by the clinical stage of infection (acute vs. middle vs. late) in the transmitting partner. The acute and late stages of HIV are found to be more infectious because of increased viral loads, but the asymptomatic stage of infection will typically contribute more to the net transmission of HIV-1 over the lifetime of an infected individual, because of its longer duration. HIV-2 has a slow progression. HIV-1 is responsible for the majority of the infection seen globally [10-12]. Other factors that affect HIV transmission include sexually transmitted diseases that can increase infection susceptibility by two- to 11-fold due to inflammation and pooling of CD4 cells. Pregnancy is associated with a greater than twofold increase in HIV acquisition risk. In contrast, studies have shown that circumcision decreased transmission acquisition risk in the male partner by 60%, attributed to the reduction of available CD4 cells [12].

Oral transmission of HIV is a rare event. Experimental studies to determine the HIV inhibitory mechanisms showed that the hypotonicity of the saliva was the major factor, causing the disruption of HIV-infected leukocytes. However, this protection was lost in the presence of other ingested isotonic fluids like milk, seminal fluid or excessive bleeding. Oral transmission to contacts or healthcare workers may increase in situations that reduce the hypotonicity of the saliva [13].

According to India’s National AIDS Control Organization (NACO), the bulk of HIV infections in India occur during unprotected heterosexual intercourse among sex workers and injection drug users. The prevalence of HIV infection in 2021 in India’s high-risk groups is as in Table 1^ ^[14].

**Table 1:** HIV prevalence in high-risk populations in India (2021) Source: UNAIDS data book, 2022 [14].

High-risk populations in India	HIV prevalence
Sex workers	1.9%
Men who have sex with men	3.3%
People who inject drugs	9%
Transgender people	3.8%
Prisoners	1.9%

Risk of Transmission for Healthcare Staff

Swedish studies on the impact of effective ART showed that it not only reduced the morbidity and mortality in the patients but also greatly reduced the risk of HIV transmission. They report no risk of transmission of HIV to staff in health and dental care from patients on effective ART who have fully suppressed viral load from splashes of body fluids in the eye and mucous membranes or superficial needle pricks. Chances of HIV transmission increase in case blood is injected or in major surgical interventions and the HIV-infected person is obliged to inform the person at risk about their HIV status [15].

A 13-year University of Pittsburgh study on HIV transmission in healthcare workers after occupational exposure concluded that “HIV does not seem to be as easily transmitted by needlestick, laceration, or splash injuries as previously surmised” [16].

Pathogenesis

Different viral and host factors determine how the HIV disease progresses in infected individuals. HIV is able to counteract the host immunity by utilizing the signal transduction pathways to connect with the cells, induce inflammation and manipulate the host defence mechanisms to survive. HIV is a highly polymorphic virus, a feature attributed to its error-prone reverse transcriptase. CD4 + receptor and chemokine receptors CCR5 and CXCR4 play an important role in the HIV life cycle. CD4 (cluster of differentiation 4) receptors are seen on dendritic cells, T cells, macrophages, and monocytes. The external glycoprotein (gp120) on the HIV-1 envelope attaches to the CD4+ receptor on the cell membrane of host cells on entry. HIV uses chemokine co-receptors (CCR5, CXCR4) after binding to the cell membrane to fuse and transfer its genetic material into the cell. With the help of the viral reverse transcriptase enzyme, viral RNA is changed to DNA. With the help of viral protein integrase and host DNA repair enzymes, the viral genome gets integrated into the host’s chromosomal DNA changing the cell into a virus producer. The replicated virions are released from the infected cell by budding from the host plasma membrane [10,17-18].

Fiebig Stages of Acute and Early HIV Infection

Fiebig and colleagues devised a laboratory staging system for acute and early infection [12, 19]:

Eclipse phase: the period between the infection of the first cell and the detection of the virus in the blood. The average duration is 10 days. The virus replicates in CD4+ T cells in the mucosa and submucosa at the portal of entry and makes its way to the lymph vessels, gut-associated lymphoid tissue (GALT) and systemic lymphatic tissues. Therefore, the virus is not detectable in the blood during this phase.

After its detection in the blood, the virus increases exponentially because of explosive replication in GALT and peripheral lymphoid tissue.

Fiebig stage I: During ramp-up viremia; HIV-1 viral RNA appears in the plasma. The average duration is seven days.

Fiebig stage II: This stage is characterized by the detection of viral p24 antigen. P24 antigen is a viral core protein that transiently appears in the blood during the ramp-up phase once HIV-1 RNA levels rise above 10,000 copies/ml. The average duration is five days.

Fiebig stage III: The virus-specific antibodies can be detectable first by recombinant protein-based enzyme-linked immunosorbent assay. The duration is three days.

Fiebig stage IV: Western immunoblotting shows indeterminant banding pattern. The average duration is six days.

Fiebig stage V: Western immunoblotting shows a diagnostic banding pattern but missing p31 reactivity. The average duration is 70 days.

Fiebig stage VI: Western immunoblotting shows a diagnostic banding pattern with p31 reactivity. The average duration is open-ended [12, 19].

Clinical Course of HIV Infection

Primary/acute infection: There is an initial burst of viremia because of systemic dissemination from lymphoid tissues and peak plasma levels of the virus reach approximately 10^6^-10^7^ copies per ml [18]. The intense inflammatory response is generated and characterized by high levels of cytokines and chemokines, a “cytokine storm”. Acute retroviral syndrome, in some individuals, is seen as fever, rash, night sweats, severe fatigue, headache, diarrhoea, pharyngitis, arthralgias and myalgias [19]. The primary phase shows high infectivity.

Clinical latency / chronic phase / established infection: Immune response develops with HIV-specific CD8+ responses and target cell limitation. After the peak, as the immune system is activated the virus plateaus to a constant level which is called a viral set point [18]. Usually presents as an asymptomatic phase as CD4+ T-cell counts are above 500 per μl [20]. Inactive HIV infection persists within memory CD4+ T cells in the lymphatic tissues (GALT) where even the host immune mechanisms may not reach. They have the potential to reactivate and produce infection later [18]. Clinical latency does not indicate disease latency. HIV disease is active and causes the reduction of infected CD4 + T cells in the lymphoid tissues even with antiretroviral treatment [10].

AIDS-defining illness/ advanced HIV disease: CD4+ T-cell counts drop below 200 per μl and there is increased susceptibility to opportunistic infections or neoplasms. The clinical picture may be characterized by severe and persistent constitutional signs and symptoms [20].

Gut-associated lymphoid tissue (GALT) which has numerous sus­ceptible memory T lymphocytes shows high HIV replication and CD4+ T cell death resulting in increased intestinal lining permeability that causes systemic trans­location of bacterial products and increased immune activation. Hyperactive immune response with the proliferation of CD4+ and CD8+ T cells and their eventual death results in progress to AIDS. The average progression period to AIDS is 8-10 years with variations such as rapid progression, slow progression and rarely, some never progress at all [18, 20].

Diagnosis and testing

Proper history and risk factors for HIV infection in the suspected individual are assessed. Any related clinical manifestations are observed. The diagnosis of HIV infection is through serological tests that detect viral antigens, anti-HIV antibodies or both. Rapid antibody tests that use blood from a fingerstick or oral fluid are also available that help in fast screening in resource-constrained settings. The antibody tests may not give accurate results during the primary stage of infection when the HIV antibodies are not formed and in children below 1.5 years who are protected by their mother’s immunoglobulins. These conditions warrant the detection of the viral RNA or p24 antigen for correct diagnosis [10].

CD4+ T cell count and viral loads are also measured to check the degree of immunodeficiency and the rate of immune destruction respectively that will indicate the disease progression/ staging. CD4+ T cells are quantified using flow cytometry. Viral loads can be measured using commercially available amplification assays [10].

Newer fourth-generation enzyme-linked immuno-assays (EIA) that combine antigen-antibody tests are currently preferred for screening as they simultaneously detect p24 antigen and anti-HIV-1/2 antibodies. In the absence of discrimination of the positive test result because of p24 antigen or HIV antibodies in asymptomatic individuals, it becomes difficult to differentiate between acute infection and established infection. For positive results, another confirmatory antibody assay to differentiate between HIV-1 or HIV-2 infections is indicated [18, 19].

Clinical manifestations

The primary stage can manifest as an asymptomatic or acute retroviral syndrome of variable severity. It usually presents with fever post-exposure, swollen lymph nodes, inflamed throat, rashes on the skin, oral and genital ulcers, and transient decrease in white blood cells which is supportive to other infections [21].

For benefit of regions with a paucity of laboratory facilities and equipment to test for HIV infection status, WHO has provided the revised clinical staging (Tables 2-3) and immunological classification of HIV. This classification is used for established cases where the clinical staging is predictive of survival and disease progression without ART [21].

**Table 2:** WHO clinical staging of established HIV infection Abbreviations: WHO, World Health Organization; HIV, human immunodeficiency virus Source: WHO case definitions of HIV for surveillance and revised clinical staging and immunological classification of HIV-related disease in adults and children, 2007 [21].

HIV-associated symptoms	WHO clinical stage
Asymptomatic	1
Mild symptoms	2
Advanced symptom	3
Severe symptoms	4

**Table 3:** WHO clinical staging of HIV/AIDS for adults and adolescents with confirmed HIV infection Source: World Health Organization 2007: WHO case definitions of HIV for surveillance and revised clinical staging and immunological classification of HIV-related disease in adults and children. World Health Organization, Geneva; 2007 [21].

WHO clinical staging of HIV/AIDS for adults and adolescents with confirmed HIV infection.	
Clinical stage 1	Asymptomatic; persistent generalized lymphadenopathy
Clinical stage 2	Moderate unexplained weight loss (<10% of presumed or measured body weight); recurrent respiratory tract infections (sinusitis, tonsillitis, otitis media and pharyngitis); herpes zoster; angular cheilitis; recurrent oral ulceration; papular pruritic eruptions; seborrhoeic dermatitis; fungal nail infections
Clinical stage 3	Unexplained severe weight loss (>10% of presumed or measured body weight); unexplained chronic diarrhoea for longer than one month; unexplained persistent fever (above 37.6°C intermittent or constant, for longer than one month); persistent oral candidiasis; oral hairy leukoplakia; pulmonary tuberculosis (current); severe bacterial infections (such as pneumonia, empyema, pyomyositis, bone or joint infection, meningitis or bacteraemia); acute necrotizing ulcerative stomatitis, gingivitis or periodontitis; unexplained anaemia (<8 g/dl), neutropenia (<0.5 × 109 per litre) or chronic thrombocytopaenia (<50 × 109 per litre)
Clinical stage 4	HIV wasting syndrome; Pneumocystis pneumonia; recurrent severe bacterial pneumonia; chronic herpes simplex infection (orolabial, genital or anorectal of more than one month’s duration or visceral at any site); oesophageal candidiasis (or candidiasis of trachea, bronchi or lungs); extrapulmonary tuberculosis; Kaposi’s sarcoma; cytomegalovirus infection (retinitis or infection of other organs); central nervous system toxoplasmosis; HIV encephalopathy; extrapulmonary cryptococcosis including meningitis; disseminated non-tuberculous mycobacterial infection; progressive multifocal leukoencephalopathy; chronic cryptosporidiosis (with diarrhoea); chronic isosporiasis; disseminated mycosis (coccidioidomycosis or histoplasmosis); recurrent non-typhoidal Salmonella bacteraemia; lymphoma (cerebral or B-cell non-Hodgkin) or other solid HIV-associated tumours; invasive cervical carcinoma; atypical disseminated leishmaniasis; symptomatic HIV-associated nephropathy or symptomatic HIV-associated cardiomyopathy

Some oral lesions act as early signals of HIV infection. Oral conditions strongly associated with HIV infection include oral candidiasis, linear gingival erythema, necrotizing ulcerative gingivitis and periodontitis, etc. They are seen in 50% to 80% of HIV-AIDS patients [22].

Antiretroviral therapy (ART)

Antiretroviral therapy (ART) / highly active ART (HAART) / combination ART (cART) is used for managing HIV infections. ART uses a set of at least three different classes of anti-retroviral medicines to break the multiplication cycle of HIV, reducing the virus to undetectable levels (Table 4) [6]. 

The drugs used in ART are not a cure for HIV but they have enabled HIV infection to become a manageable chronic disease. They cause viral suppression allowing CD4 counts to improve and thus decrease immunodeficiency. ART has been able to suppress opportunistic infections and reduce the risk of transmission effectively in HIV-infected patients allowing them to have a normal life [6, 10, 22]. 

**Table 4:** Goals of anti-retroviral therapy Source: National AIDS Control Organization (2021), Government of India [6].

Goals of Anti-Retroviral Therapy	
Clinical goals	Increased survival and improvement in quality of life
Virological goals	Greatest possible sustained reduction in viral load
Immunological goals	Immune reconstitution, that is, both quantitative and qualitative
Therapeutic goals	Rational sequencing of drugs in a manner that achieves clinical, virological and immunological goals while maintaining future treatment options, limiting drug toxicity and facilitating adherence
Preventive goals	Reduction of HIV transmission by suppression of viral load

Classification of ART Therapy Drugs

Based on the step of the virus cycle that is inhibited, they can be grouped into various classes [6, 18]: 

Entry inhibitors: These drugs stop HIV from entering the cell.

Nucleoside/nucleotide reverse transcriptase inhibitors (NRTI): These are nucleoside/ nucleotide analogues which act by getting preferentially incorporated into the DNA of the virus, thereby stopping the DNA synthesis.

Integrase strand transfer inhibitors: They prevent the host and the HIV DNA from attaching by blocking the integrase enzyme.

Non-nucleoside reverse transcriptase inhibitors (NNRTIs): These drugs bind to reverse transcriptase enzyme causing a conformational change of the enzyme and inhibition of reverse tran­scription from RNA to DNA.

Protease inhibitors: Protease enzyme helps in the conversion of immature viral particles to mature virus. Protease inhibitors block this conversion.

The classes and names of anti-retroviral drugs are listed in Table 5 [6, 18]. 

**Table 5:** Classes of anti-retroviral drugs Source: National AIDS Control Organization (2021). National Guidelines for HIV Care and Treatment, Government of India [6].

Class of Anti-Retroviral drug	Names of Anti-Retroviral drug
Nucleoside reverse transcriptase inhibitors (NsRTI)	Zidovudine (AZT), Stavudine (d4T), Lamivudine (3TC), Abacavir (ABC), Didanosine (ddl), Zalcitabine (ddC), Emtricitabine (FTC)
Nucleotide reverse transcriptase inhibitors (NtRTI)	Tenofovir Disoproxil Fumarate (TDF), Tenofovir Alafenamide (TAF)
Fusion inhibitors (FI)	Enfuvirtide (T-20)
Non-nucleoside reverse transcriptase inhibitors (NNRTI)	Nevirapine (NVP), Efavirenz (EFV), Delavirdine (DLV), Rilpivirine (RPV), Etravirine (ETV), Doravirine (DOR)
Integrase Inhibitors	Dolutegravir (DTG), Raltegravir (RGV), Elvitegravir (EVG), Bictegravir (BIC), Cabotegravir (CAB)
CCR5 entry inhibitor	Maraviroc (MVC)
Protease inhibitors (PI)	Saquinavir (SQV), Ritonavir (RTV), Nelfinavir (NFV), Amprenavir (APV), Indinavir (INV), Lopinavir (LPV), Fosamprenavir (FPV), Atazanavir (ATV), Tipranavir (TPV), Darunavir (DRV)
Post-attachment maturation inhibitor	Ibalizumab (IBA)

Classes of Anti-Retroviral Drugs

Current protocols support the view that all HIV-infected patients should be given ART irrespective of their clinical stage, CD4 count, age or risk [6]. ART is not always fully effective. Drug resistance remains a major concern in cases of laxity and irregularity in using ART. When HIV multiplies in the presence of antiretroviral drugs, the selection of drug resistance-inducing mutations occurs. Drug resistance can be transmitted to others. All classes of anti-retroviral drugs show adverse effects and toxicities. These drugs must be administered for life so subtle toxicities can have an additive effect and reduce the quality of life in PLHIV. Some adverse effects of ART include renal toxicity, hepatic toxicity, metabolic abnormalities, reduced haemoglobin levels, decreased neutrophil count, bone marrow suppression, GI intolerance, headache, insomnia, myopathy, lactic acidosis, skin and nail hyperpigmentation, xerostomia, neurological issues, drug interactions, etc. [18].

Xerostomia because of ART causes disturbance in normal oral microflora facilitating the colonization of atypical microorganisms, which aggravates oral pathologies like dental caries, and periodontitis. There is a loss of oral structure and function that promote malnourishment and immune dysregulation [22].

Orthodontic considerations

Changes in oral health care primarily based on HIV status are not supported. Nevertheless, individual assessment of each patient is required as HIV disease can present with a variety of oral and systemic clinical effects [23].

PLHIV visiting an orthodontic clinic can be grouped into three categories: (a) Patients who don’t know they are HIV positive; (b) HIV-positive but do not wish to disclose their HIV status to the orthodontist; (c) they know their HIV status and disclose it voluntarily.

Since the possibility of exposure to unknown HIV patients is present, a general case history form that has a provision for a comprehensive assessment of the patient's health is needed. It helps to screen for medical problems, assess the risks to the patient associated with the provision of orthodontic treatment, and evaluate conditions and diseases that may necessitate modification of orthodontic treatment. Treatment should be prioritized to include alleviation of pain, restoration of function, prevention of further disease and consideration of esthetics [23,24].

HIV infection is not a contraindication for orthodontic treatment. Patients with undiagnosed HIV infection have received successful orthodontic treatment, suggesting that asymptomatic HIV-infected patients respond to orthodontic treatment in the same manner as non-infected orthodontic patients. Patients’ overall health, stage of disease, presence of other co-infections and co-morbidities and willingness to cooperate influence the treatment planning [23-25].

Pointers in Orthodontic Treatment for PLHIV

Unknown/undisclosed HIV-positive status: The practitioner should take a comprehensive patient history that can assess risk factors or risky behaviour for HIV infection. For asymptomatic patients, regular orthodontic treatment can be considered by employing standard precautions and infection control procedures. Treatment is deferred in the case of symptomatic patients. The patients should be advised to undergo HIV testing or to refer to a physician/designated HIV centre for suspected cases showing clinical indicators. Clinical symptoms can range from unexplained weight loss to lymphadenopathy to severe issues like Kaposi's sarcoma, opportunistic infections etc. as classified by WHO [21].

Known HIV-positive status: For symptomatic patients, orthodontic treatment can be delayed till symptoms reduce and other systemic and oral conditions are treated. For asymptomatic patients, it is necessary to first assess if the patient is under ART. Those who are not on ART should be referred for the treatment of HIV. Coordination with the patient’s general physician will help in customizing treatment to fit the patients' health status. Certain tests may be advised to know patients' health status like CD4 counts for the level of immunodeficiency. The CD4 count should be > 500 per μl. Viral load to ascertain the potential transmissibility of HIV infection should be < 1000 copies/ml. Platelet count, bleeding and clotting time should be checked to look for bleeding tendency, which is a side effect of ART, especially if extraction treatment is planned.

A low absolute neutrophil count indicates susceptibility to infections. So based on the need, prophylactic or post-treatment, antibiotic therapy may be prescribed. If antibiotic therapy is required, drug interactions with anti-retroviral drugs or other medications that are used should be considered. Antibiotic prophylaxis is not mandatory for all HIV-positive patients [24].

Standard precautions and infection control procedures should be implemented and screening for any developing oral manifestations should be done periodically. Xerostomia can occur due to ART. Artificial salivary substitutes can be given to manage the condition and additionally, fluoride supplementation can be prescribed to prevent caries from dry mouth. Chlorhexidine mouth rinses should be advised for oral hygiene maintenance.

General orthodontic planning should include minimizing blood exposure, conservative and short duration of treatment (non-extraction treatment if possible), or less frequent appointments, removable appliances to permit oral hygiene and reduce gingival inflammation, emphasis on good oral hygiene and meticulous care of retainers and appliances [24]. Bonded attachments can be preferred instead of bands during fixed appliance treatment and the use of stainless steel ligatures can be reduced to prevent ligature pricks. Studies to ascertain the prevalence rate of percutaneous injuries in orthodontists in the United States revealed less than one injury per orthodontist per year. The majority of the injuries happened outside the mouth, during archwire changes and involved the index finger and thumb. The rate of injury was less among practitioners with more experience [26]. Sharp attachments/appliance surfaces and sharp wire ends should be avoided to prevent nicks and cuts in the patients' oral cavity as well as to the clinician. The use of self-ligating brackets will be beneficial in avoiding steel ligature ties [27]. The above guidelines are not absolute and usually, the treatment has to be customized according to the patients' individual requirements. 

As the lymphoid tissue is the seat of HIV replication and spread, adenoid hypertrophy is commonly seen in HIV-infected adults and children. Adenoid hypertrophy can result in obstruction of the airway and mouth breathing. In growing children, mouth breathing causes vertical growth of the face, lip hypotonicity, snoring, and increased risk of pneumonia [28-30].

Removable and fixed orthodontic appliances are associated with increased Candida colonization, which is attributed to the fact that these appliances disturb the oral microflora. Removable appliances are made with polymethylmethacrylate (PMMA) material, which has porosities due to the release of residual monomer after polymerization. These porosities provide areas forCandida to adhere to and form a biofilm. Decreased salivary pH, improper salivary clearance of the biofilm at the acrylic-mucosa interface and microtears in the mucosa from the appliance wear allows fungi to enter the mucosa, causing infection. In the case of fixed orthodontic appliances, Candida colonization is related to increased plaque-retentive areas. Composite and ceramic brackets show greater colonization of fungi because of the porosity and roughness of the material compared with metal brackets. Increased Candida colonization does not mean infection. The ability to cause infection is associated with the host's immune response. Candida albicans is the most prevalent species seen in the general population but in HIV-infected patients, a larger number of non-albicans species are detected. HIV-infected individuals are more susceptible to Candida infection because of their immune deficiency. This problem is exaggerated by orthodontic therapy. Therefore, the use of newer less porous thermoplastics in removable appliances, and good oral hygiene maintenance will reduce Candida adhesion and colonization [31-34].

Studies in vertically transmitted HIV-positive children and adolescents showed a higher prevalence of molar incisor hypomineralization (MIH) probably associated with systemic conditions seen in HIV infection or ART regimens using protease inhibitors. Delayed bone development and delayed dental eruption were also reported despite the use of ART. Although there was a positive association between delayed dental eruption and ART, a causal relationship has not been established conclusively. Hypomineralization of the teeth affects the bracket bond strength. A 5% sodium hypochlorite (NaOCl) has been used to deproteinize the enamel before bonding to improve the bond strength. The use of 2% sodium fluoride (NaF) has been suggested on hypomineralized teeth before bonding to reduce microleakage under the brackets [35-40].

One study noted increased tooth wear in HIV-infected individuals on ART. Tooth wear was not directly caused by ART but rather related to problems like bruxism, psychological issues and psychiatric medications. A night guard is suggested for preventing tooth wear [41].

ART causes various metabolic abnormalities. Lipodystrophy is one such common issue and presents in different forms. Fat accumulation type (lipohypertrophy), fat loss type (lipoatrophy) and a mixed type. Pithon et.al. reported a case of an orthodontic patient on ART who developed lipoatrophy of the face and doubted it to be because of the orthodontic treatment. Such coincidences become a problem if the correct aetiology is not deduced. Orthodontists must be aware of the side effects of ART as they have the potential to affect facial esthetics and orthodontic treatment [42].

A Brazilian study to assess the craniofacial morphology of HIV-infected children on ART (study group) showed no significant differences compared with matched non-HIV-infected children (control group) in the ages of 6-17 years. Increased maxillary rotation in the 6-8 year age group and reduced SNA (sella, nasion, A point) angle in the 13-17 year age group were the only significant values in the study group [43].

Cervical vertebrae maturation was correlated with age in HIV-infected and uninfected children in a study and it was found that a weak correlation existed between cervical vertebrae maturation and age in HIV-infected children. The authors suggest caution in using cervical vertebrae maturation for predicting skeletal development in HIV-positive children for orthopaedic and orthodontic procedures [44].

In AIDS/advanced HIV infection, orthodontic treatment is contraindicated as treating opportunistic and other co-morbid conditions take precedence.

Standard precautions

Considering the above possibilities it is important that in orthodontic practice every patient should be considered potentially infectious. Standard precautions are the basic infection control procedures that should be undertaken in all healthcare settings no matter the suspected or confirmed infection status of the patient. They are a stop to the transmission of infections in healthcare situations by protecting the healthcare workers from contracting infections and preventing the healthcare workers from spreading infections to the patients [45].

Concise steps in standard precautions according to the Centers for Disease Control and Prevention (CDC), USA include: (1) antisepsis of hands; (2) use of gloves, masks, eyewear, face shields (PPA: personal protective equipment); (3) respiratory hygiene/cough etiquette; (4) safety protocols for working with sharp objects and their disposal; (5) safe injection practices; (6) sterilization of instruments, equipment and devices; (7) disinfection of surfaces and surrounding environments [45].

Post-exposure prophylaxis (PEP)

Healthcare providers are prone to occupational exposure to infected material at work. Despite the implementation of standard precautions, accidental exposure to HIV-infected fluids or tissues (Table 6) can happen. Exposure can occur from percutaneous injury (needle stick injury or cut from sharp object), with 0.3% frequency for HIV, through the mucosa of the eye or mouth (0.09% for HIV) and through exposed skin. 

First aid should be given immediately after the injury: wounds and skin sites exposed to blood or body fluids should be washed with soap and water, and mucous membranes flushed with water [6, 46].

**Table 6:** Risk of exposure from different body fluids Source: National AIDS Control Organization (2021). National Guidelines for HIV Care and Treatment, 2021. New Delhi: NACO, Ministry of Health and Family Welfare, Government of India [6].

Exposure to Body Fluids Considered 'at Risk'	Exposure to Body Fluids Considered 'Not at Risk,' Unless these Fluids Contain Visible Blood	
Blood, Semen, Vaginal secretions, Cerebrospinal fluid, Synovial, pleural, peritoneal, pericardial fluid, Amniotic fluid, Other body fluids contaminated with visible blood	Tears, Sweat, Urine and faeces, Saliva, Sputum, Vomitus	Unless these secretions contain visible blood

The need for PEP is decided by a scoring system that takes into account the type of exposure, whether the source is suspected or confirmed positive for HIV, and the stage of HIV infection. PEP is effective if taken immediately or within 72 hours of exposure [6, 24, 46]. PEP must be taken for four weeks (28 days). PEP is started after taking informed consent. Psychological support and counselling are provided so that adherence to drug protocol is maintained. Current PEP protocols followed in India are shown in Table 7 [6].

**Table 7:** Recommended PEP regimens in India Abbreviations: FDC, fixed dose combinations; OD, once every day (omni die), BD, twice a day (bis in die) Source: National AIDS Control Organization (2021). National Guidelines for HIV Care and Treatment, Government of India [6].

Exposed Person	Preferred Regimen for PEP Drugs and Dosages	Alternate Regimen (If the Preferred Regimen is not available or Contraindicated)
Adolescents and Adults (>10 years of age and >30 kg weight)	Tenofovir (300 mg) + Lamivudine (300 mg) + Dolutegravir (50 mg) (FDC: One tablet OD)	Tenofovir (300 mg) + Lamivudine (300 mg), (FDC: One tablet OD) + Lopinavir (200 mg)/Ritonavir (50 mg) (two tablets BD), OR, Tenofovir (300 mg) + Lamivudine (300 mg) + Efavirenz (600 mg), (FDC: One tablet OD)

After exposure, HIV testing is done to establish a ‘baseline’ for comparing later results. Post-exposure follow-up is done with or without PEP to check for possible infections and to provide psychological support. The number of positive conversions after six months is negligible so no further test is advised after a negative test at six months [6, 24, 46].

Anyone with PEP requirements should be evaluated for signs of positive HIV infection within 3-6 weeks of exposure. During the phase of PEP blood donation, breastfeeding and pregnancy should be avoided and measures to prevent cross-infection should be followed. Counselling on adverse drug reactions and on the necessity to complete the PEP regimen is provided [6].

## Conclusions

The probability of transmission of HIV is still potent as there is no cure or vaccine. Progress in anti-retroviral drugs has greatly reduced the transmission rates and improved the quality of life of PLHIV. Therefore, PLHIV coming for orthodontic treatments will increase. It becomes imperative that cross-infections between orthodontists, patients and staff are eliminated for the safety of all concerned. This mandates proper knowledge of the HIV disease and its management. Following the current protocols for infection control cannot be emphasized enough.
